# A randomized clinical efficacy trial of a psychosocial intervention to strengthen self-acceptance and reduce HIV risk for MSM in India: study protocol

**DOI:** 10.1186/s12889-018-5838-2

**Published:** 2018-07-18

**Authors:** Matthew J. Mimiaga, Beena Thomas, Kenneth H. Mayer, Kristen S. Regenauer, Alpana Dange, C. Andres Bedoya, Shruta Rawat, Vinoth Balu, Conall O’Cleirigh, Katie B. Biello, Vivek Anand, Soumya Swaminathan, Steven A. Safren

**Affiliations:** 1 Departments of Behavioral & Social Health Sciences and Epidemiology, Brown University School of Public Health, Providence, RI USA; 2 Center for Health Equity Research, Brown University, Providence, RI USA; 3 The Fenway Institute, Fenway Health, Boston, MA USA; 4 Department of Psychiatry & Human Behavior, Alpert Medical School, Brown University, Providence, RI USA; 5 National Institute for Research in Tuberculosis (NIRT), Chennai, India; 6 Harvard Medical School, Boston, MA USA; 7 Beth Israel Deaconness Medical Center, Boston, MA USA; 8 Department of Global Health and Population, Harvard T. H. Chan School of Public Health, Boston, MA USA; 9 Behavioral Medicine Program, Department of Psychiatry, Massachusetts General Hospital, Boston, MA USA; 10 The Humsafar Trust, Mumbai, India; 11 Department of Psychology, University of Miami, Coral Gables, FL USA

**Keywords:** HIV prevention, STI prevention, MSM, Self-acceptance, Psychosocial intervention, Randomized controlled trial, India

## Abstract

**Background:**

Men who have sex with men (MSM) in India are a key group at risk for HIV acquisition and transmission. They are also an extremely marginalized and stigmatized population, facing immense psychosocial stressors including, but not limited to, stigma, homophobia, discrimination, criminalization, low self-esteem, low self-acceptance, distress, and, as a result, high rates of mental health problems. Although these multi-level psychosocial problems may put MSM at high risk for HIV acquisition and transmission, currently HIV prevention interventions in India do not address them. This paper describes the design of a psychosocial intervention to reduce HIV risk for MSM in India.

**Methods:**

Funded by the National Institute of Mental Health, this study is a two-arm randomized clinical efficacy trial of a self-acceptance based psychosocial HIV prevention intervention, informed by the minority stress model and syndemic theory, that was developed with extensive community-based formative work and input from the Indian MSM community and key informants who are knowledgeable about the experiences faced by MSM in India. Participants are MSM in Chennai and Mumbai who endorsed recent sexual behaviors placing them at high risk for HIV/sexually transmitted infection (STI) acquisition and transmission. Enrolled participants are equally randomized to either 1) the experimental condition, which consists of four group and six individual counseling sessions and includes standard of care HIV/STI testing and counseling, or 2) the standard of care condition, which includes HIV/STI testing and counseling alone. The primary outcomes are changes in the frequency of condomless anal sex acts and STI incidence (syphilis seropositivity and urethral, rectal, and pharyngeal gonorrhea and chlamydia infection. Major study assessment visits occur at baseline, 4-, 8-, and 12-months.

**Discussion:**

HIV prevention interventions that address the psychosocial stressors faced by MSM in India are needed; this study will examine the efficacy of such an intervention. If the intervention is successful, it may be able to reduce the national HIV/AIDS burden in India while empowering a marginalized and highly stigmatized group.

**Trial registration:**

ClinicalTrials.gov Identifier: NCT02556294, registered 22 September 2015.

## Background

India is home to an estimated 2.1 million people living with human immunodeficiency virus (HIV), nearly 6% of all people living with HIV in the world [1]. With 4.3% of men who have sex with men (MSM) in India living with HIV, UNAIDS has identified this population as a key group at risk for HIV acquisition and transmission [1, 2]. MSM in India may also be a ‘bridge’ population, spreading HIV to other populations, since many MSM marry and engage in unprotected intercourse with their wives, while still partaking in risky sexual behavior with men [3, 4].

MSM in India are extremely marginalized and stigmatized, and face many sources of psychosocial stress including stigma, homophobia, discrimination, and even criminalization [5]. While MSM sexual activity is prevalent in the country, such sexual behavior is taboo [3, 6] and illegal [7], influencing the MSM community to be largely secretive and silent, and placing them at risk of abuse. Prior work in India has found high levels of perceived stigma, harassment from police and “thugs,” and negative interactions with healthcare providers among MSM, and suggests that Indian MSM face a variety of psychosocial problems including low self-esteem, internalized homophobia, difficulty in coming out to others, family pressure to marry and have children, leading a “double life” (being in a relationship with a woman, despite having a same-sex sexual orientation), difficulty disclosing one’s MSM status to one’s female partner or spouse, relationship problems with male sexual partners, and, for those who are HIV-positive, disclosing and coping with one’s HIV status [8]. Furthermore, due to such multi-level psychosocial stressors, this population may also experience high rates of psychiatric disorders; a mental health study in Mumbai, India identified major depressive episodes (29%), anxiety disorders (24%), and suicidal ideation (45%) among MSM, each of which were independently associated with sexual risk behavior that places men at greater risk of acquiring HIV [9, 10]. Among MSM in the United States (US) [11–13] and other regions of the world [14, 15] it has been shown that co-occurring “syndemics” of psychosocial health problems accelerate HIV acquisition among MSM. Due to the extremely stigmatizing cultural context and subsequent high rates of mental health problems, MSM in India may experience even more psychosocial distress than MSM in other regions of the world, which may further potentiate HIV spread in this group [10, 16].

Despite the high psychosocial stress, current HIV prevention interventions for MSM in India are primarily focused on condom distribution and HIV education, but do not address the many psychosocial problems that impact this population and may increase HIV risk. Yet, internationally, HIV prevention interventions in MSM that are based on theoretical models, span multiple sessions, and include interpersonal skills training and multiple delivery methods have been shown to be effective [17, 18]. Thus, with extensive community-based formative work and input from the Indian MSM community and their providers [8, 10, 16, 19–29], we designed a psychosocial intervention to increase self-acceptance and reduce HIV acquisition and transmission risk among MSM in India.

### Study objectives

The primary aim of this study is to examine the efficacy of the psychosocial intervention with standard of care (standard HIV/sexually transmitted disease (STI) counseling and testing) in comparison to just the standard of care on [1] changes in frequency of condomless anal sex acts, and [2] STI incidence. Secondary aims are [1] to examine the efficacy of the psychosocial intervention on potential mediators, such as self-acceptance, self-esteem, and distress, and (b) to assess the incremental cost-effectiveness of the psychosocial intervention relative to standard of care.

## Methods/design

### Study design

**Fig. 1:**
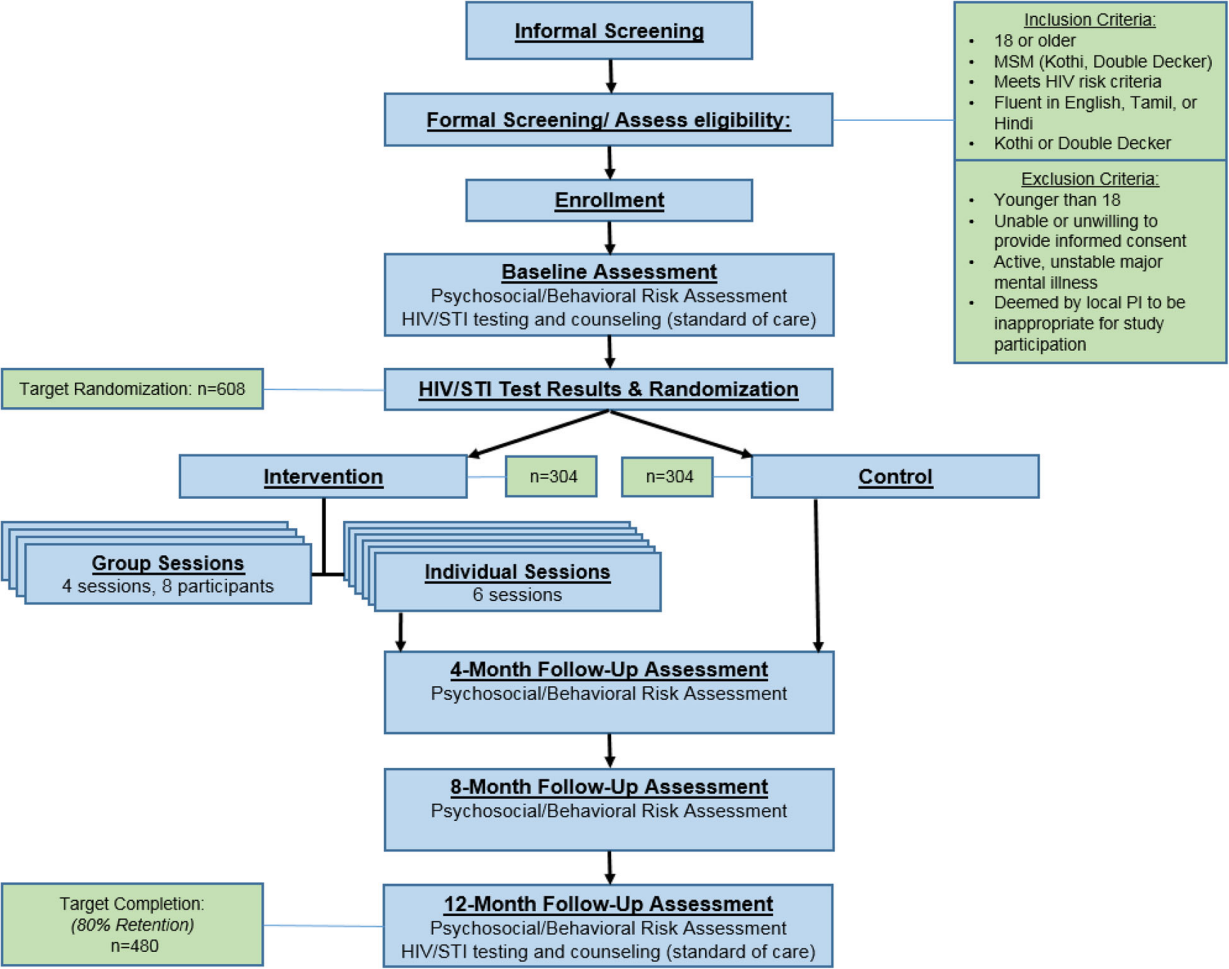
Flow chart of study intervention process

The study is a two-arm randomized clinical trial of a self-acceptance based psychosocial HIV prevention intervention designed for MSM in Chennai and Mumbai, India. Approximately 608 participants are randomized to either the psychosocial intervention (with standard of care) condition or solely to the standard of care (control) condition. Participants in both arms complete assessments at four timepoints: baseline (psychosocial assessments and HIV/STI testing), post-intervention (psychosocial assessments only), 8-month follow-up (psychosocial assessments only), and 12-month follow-up (psychosocial assessments and HIV/STI testing). The study flow is demonstrated in Fig. 1.

### Participants

#### Eligibility criteria

Participants are MSM living both with and without HIV in Chennai and Mumbai, India. To be eligible for the study, participants must be: (a) aged 18 or older; (b) a man who has sex with men; (c) a Kothi or Double Decker MSM subtype; (d) fluent in English, Tamil, or Hindi; and (e) at risk for acquisition of HIV (defined by any of the following in the four months prior to screening: protected or unprotected anal sex with four or more male partners, a diagnosis of a STI, a history of transactional sex activity, or condomless anal sex with a man who was HIV unknown status or serodiscordant). Participants are excluded if they are under 18 years old; are unable or unwilling to provide informed consent; have an active, untreated, unstable major mental illness (i.e., untreated psychosis or mania) that would interfere with treatment or any primary psychotic disorder, even if treated; or are deemed by the local PI or study outreach staff to be inappropriate for participation in the study (e.g., engaging in deception about eligibility).

Instead of being categorized under one identity (e.g. “gay”), MSM in India tend to be categorized by behavior and sex role. Subgroups include *Kothi* (feminine acting/appearing, predominately receptive partners in anal sex), *Panthi* (masculine appearing, predominately insertive partners in anal sex), and *Double Deckers* (both insertive and receptive in anal sex) [3, 30, 31]. Based on input from the Indian community members and colleagues, Kothi and Double Decker MSM were deemed most likely to benefit from the intervention, as they tend to be the most stigmatized and harassed. Prior research suggests that they would prefer not to have an intervention with Panthi MSM or other MSM identities; thus, the study is only open to Kothi and Double Decker MSM.

#### Sample recruitment procedures

In both cities, recruitment efforts are led by non-governmental organizations (NGOs) with extensive experience working with and recruiting MSM. These NGOs already follow thousands of MSM in programs not associated with the study, and have previously mapped out where MSM meet, including “cruising” sites. The NGOs recruit participants for this study via word-of-mouth and extension of current outreach efforts. All recruiters are aware of local opinions regarding sexuality, and recruit discreetly.

#### Randomization

Approximately half of participants are randomized to the psychosocial intervention condition (*N* = ~ 300 across both sites; ~ 150 per study site) and approximately half are randomized to the standard of care control condition (*N* = ~ 300 across both sites; ~ 150 per study site). Random assignments are generated by an interactive web-based response system that can be monitored across study sites. Those randomized to the psychosocial intervention are assigned in order of their randomization into batches of 8 participants for the purpose of doing group intervention sessions together. All batches, except potentially the final batch, are expected to have 8 participants.

### Procedure

#### Psychosocial intervention

The psychosocial intervention focuses on self-acceptance/self-esteem as a central protective resilience factor fostering HIV-related self-care and decreasing mental-health related distress for MSM in India [21]. Informed by community work spanning a decade [22], the intervention acknowledges many psychosocial problems for MSM in India across many levels, including community, family, and individual; but those who appear to be most resilient also have high self-acceptance. The intervention additionally places topics within the unique context of Indian MSM. We hypothesize that emphasizing self-acceptance in an HIV prevention intervention will increase self-care and decrease distress among MSM in this setting. Participants randomized to the psychosocial intervention receive four group counseling sessions and six individual counseling sessions that are conducted in either Hindi or Tamil, in addition to the standard of care (see below).

Each group session lasts approximately two hours and focuses on fostering self-acceptance in participants through developing skills, knowledge, and confidence in their own ability to overcome obstacles in their lives. Sessions begin with participants sharing one proud moment that occurred in the previous week, and feature a variety of interactive activities, including discussions, videos, games, and role-plays. The themes of the group sessions are: [1] self-acceptance: barriers and strategies, and coping with pressures from family and society; [2] safety and self-care: safer ways to meet men, and alcohol and substance use and misuse; [3] health risk: HIV education and STI vulnerability; and [4] tying it all together: risk reduction skills and looking to the future.

The purpose of individual sessions is to provide additional support for participants struggling with issues such as disclosure of their MSM or HIV status, case management, or linkage to additional care. Each session lasts approximately one hour, and participants are free to discuss topics that they are not comfortable addressing in front of a group. The themes of the individual sessions are: [1] self-esteem and coming out experience; [2] risk limits; [3–5] risk education, risk reduction, and problem solving; and [6] relapse prevention and wrap up/exit interview.

#### Standard of care

Participants in both conditions receive the standard of care HIV/STI testing and counseling at their baseline and 12-month follow-up assessments. HIV/STI testing occurs in a lab associated with the study site. Specifically, participants are tested for HIV, syphilis, chlamydia, and gonorrhea.

In addition to the HIV/STI testing, participants also receive a pre-test and post-test HIV counseling session, as is standard practice in India. At counseling sessions, risky behaviors, self-perception of risk, barriers to changing the risky behaviors, and support plans in the case of an HIV-positive result are discussed. Participants are also given educational materials on topics such as Hepatitis B, C, and D, HIV infection, STIs, overdose prevention/ first aid at overdose, drug use harm reduction, safe sex, and contraception.

#### Study assessments.

The study is comprised of four main assessments: baseline, 4-month follow-up, 8-month follow-up, and 12-month follow-up. All assessments contain self-report questionnaires, filled out with the help of a blinded independent interviewer on a tablet. To minimize the social desirability bias that may occur when an interviewer is present, each major assessment visit also includes an audio computer assistant self-interview (ACASI) component without an interviewer present to measure sensitive sexual behavioral data. HIV/STI testing also occurs at the baseline and 12-month follow-up assessments.

##### Primary outcomes

[1] Changes in condomless anal sex measured with self-report using ACASI from baseline to 4-, 8-, and 12-month follow-up assessments. Based on a previous study [32, 33], participants are asked to estimate their number of sexual partners by HIV status, sex, and number of times having anal intercourse with and without a condom in the past 4 months. [2] STI incidence, measured through HIV/STI testing at baseline and the 12-month follow-up assessment. To test for HIV and syphilis, the participant has a small amount of blood drawn. HIV screening is done by rapid card tests using serum or plasma, and syphilis screening is done using serum by the Rapid Plasma Reagin (RPR) assay or the venereal disease research laboratory (VDLR) test. Additionally for syphilis, nonspecific and positive tests (RPR) are confirmed by specific tests (Microhemagglutination Assay for Treponema pallidum Antibodies (MHA-TP) or fluorescent treponemal antibody absorption (FTA-ABS)) and titers of prior tests and treatment histories are obtained in order to ensure that positive results indicate incident infections. To test for chlamydia and gonorrhea, participants provide a small urine sample for testing by nucleic acid amplification testing (NAAT), and a lab employee takes an oropharyngeal sample and a rectal swab using standard assays. All positive cases of STIs are treated on site, and anyone who tests positive for HIV is referred for outside treatment where treatment is provided free of charge.

##### Secondary outcomes

Self-acceptance and self-esteem are assessed by asking the degree to which (scale 1–10) “you accept yourself,” [19, 34] the 10-item Rosenberg scale, which uses a four-point Likert scale and has previously been used in India [35, 36], and the Collective Self-Esteem Scale (CSES), a 16-item measure that assesses thoughts and feelings associated with one’s social group on a 7-point Likert scale [37]. Distress is assessed through the Center for Epidemiological Studies Depression Scale (CES-D) [38], which has been used in India, along with the Primary Care Post Traumatic Stress Disorder Screen. Culturally-relevant sexual minority stress questions, such as the frequency and experiences of harassment, violence, losing jobs and friends, and family and social discrimination in both childhood and adulthood are also assessed. For the cost-effectiveness analysis, resource utilization data for the delivery of the intervention is collected, including the time spent by all staff preparing for and on the intervention.

### Data analyses

#### Preliminary analyses

The distribution of all variables will be assessed, including correlations between variables and the primary/secondary outcomes. Patterns of missing data will be assessed, and attrition effects will be evaluated by testing whether systematic difference exist between study completers and non-completers. Key baseline characteristics will be examined between the intervention and control conditions to ensure they are balanced; if they are not, subsequent analyses will assess whether these characteristics are confounders of the intervention.

#### Primary analyses

In the primary analysis, 1) changes in the frequency of condomless anal sex from baseline to the 4-, 8-, and 12-month follow-ups and 2) incident STIs from baseline to the 12-month follow-ups between the intervention and control conditions will be compared. Generalized linear models with link functions, selected based on the distribution of the dependent variable, will be used to analyze longitudinal data. To account for repeated measures of the outcome [39], the generalized linear models will be estimated using generalized estimating equations with robust standard error estimates. All statistical tests will determine significance with an alpha level of 0.05. To reduce the threat of bias, the intention-to-treat principle will be utilized, where individuals will be analyzed according to the condition to which they were randomized, regardless of their fidelity to the condition.

#### Secondary analyses

If the psychosocial intervention shows a differential decrease in the frequency of condomless anal sex and/or STI incidence, the extent to which this relationship works through possible mediators, such as self-acceptance, self-esteem, and distress, will be evaluated. Specifically, if a possible mediator is significantly changed by the psychosocial intervention, a mediation analysis will be conducted to determine if the effect of the intervention on the primary outcome was through the possible mediator. A cost-effectiveness analysis, examining the clinical benefits and costs of the psychosocial intervention, in comparison to the clinical benefits and costs of the standard of care control, will also be conducted.

#### Power considerations and sample size calculations

The power analysis for the primary aim is based on detecting a 30% (or greater) difference in 12-month STI incidence and sexual risk (number of unprotected anal sex acts in the past 4 months, baseline to 12-month follow-up) between the intervention and control. Based on this data, with an alpha level of 0.05 and a power of 90%, 480 study completers are needed. Assuming an attrition rate of 20% between the baseline assessment and 12-month follow-up, we need 600 study completers. However, in an attempt to have approximately 8 participants in each batch, our goal is to randomize 608 participants.

## Discussion

In this paper, we have described a two-arm randomized clinical trial of a psychosocial intervention to reduce the frequency of condomless anal sex and STI incidence in MSM in India. Currently, HIV prevention efforts for MSM in India are comprised of condom distribution and HIV education, and do not address the various psychosocial problems that this population faces. However, we hypothesize that an HIV prevention program that addresses both mental health (i.e. self-acceptance) and health behavior (i.e. condomless anal sex) simultaneously will be more successful than the current model, as both mental health problems and ‘risky’ health behavior tend to co-occur with populations at high risk for contracting HIV. This is the first HIV prevention intervention to address psychosocial factors in this population in a randomized clinical trial powered to examine efficacy. As MSM are a key at risk population for HIV acquisition and transmission in India, a successful intervention could decrease the burden of HIV/AIDS in India while simultaneously empowering a marginalized population.

## Abbreviations

CES-D: Center for Epidemiological Studies Depression Scale
CSES: Collective Self-Esteem Scale
HIV: Human immunodeficiency virus
MSM: Men who have sex with men
NAAT: Nucleic acid amplification testing
NGO: Non-Governmental Organization
RPR: Rapid Plasma Reagin
STI: Sexually transmitted infection
US: United States of America
